# Dosimetric verification of radiotherapy treatment planning systems in Serbia: national audit

**DOI:** 10.1186/1748-717X-7-155

**Published:** 2012-09-12

**Authors:** Laza Rutonjski, Borislava Petrović, Milutin Baucal, Milan Teodorović, Ozren Čudić, Eduard Gershkevitsh, Joanna Izewska

**Affiliations:** 1 Institute of oncology of Vojvodina, Sremska Kamenica, Serbia; 2 North Estonia Regional Hospital, Tallinn, Estonia; 3 International Atomic Energy Agency, Vienna, Austria

**Keywords:** Treatment planning systems, Quality assurance, Dose calculation algorithms

## Abstract

**Background:**

Independent external audits play an important role in quality assurance programme in radiation oncology. The audit supported by the IAEA in Serbia was designed to review the whole chain of activities in 3D conformal radiotherapy (3D-CRT) workflow, from patient data acquisition to treatment planning and dose delivery. The audit was based on the IAEA recommendations and focused on dosimetry part of the treatment planning and delivery processes.

**Methods:**

The audit was conducted in three radiotherapy departments of Serbia. An anthropomorphic phantom was scanned with a computed tomography unit (CT) and treatment plans for eight different test cases involving various beam configurations suggested by the IAEA were prepared on local treatment planning systems (TPSs). The phantom was irradiated following the treatment plans for these test cases and doses in specific points were measured with an ionization chamber. The differences between the measured and calculated doses were reported.

**Results:**

The measurements were conducted for different photon beam energies and TPS calculation algorithms. The deviation between the measured and calculated values for all test cases made with advanced algorithms were within the agreement criteria, while the larger deviations were observed for simpler algorithms. The number of measurements with results outside the agreement criteria increased with the increase of the beam energy and decreased with TPS calculation algorithm sophistication. Also, a few errors in the basic dosimetry data in TPS were detected and corrected.

**Conclusions:**

The audit helped the users to better understand the operational features and limitations of their TPSs and resulted in increased confidence in dose calculation accuracy using TPSs. The audit results indicated the shortcomings of simpler algorithms for the test cases performed and, therefore the transition to more advanced algorithms is highly desirable.

## Background

Quality Assurance (QA) in radiotherapy treatment planning process is essential to ensure that the dose calculation is performed correctly and to minimize the likelihood of accidental exposure [1,2]. Many studies have been performed that lead to the development of guidelines and protocols for QA of 3D radiotherapy TPSs [3,4]. Some studies have been done for solving specific problems associated with TPSs performance and dose calculation [5-7]. For the purpose of acceptance testing, commissioning and QA of TPSs, the IAEA has published Technical Reports Series No. 430 [8] that provides the general framework and describes a large number of tests and procedures to be considered by the TPS users. However, small hospitals with limited resources or large hospitals with high patient load and limited staff are not always able to perform all procedures recommended in this report, therefore the IAEA prepared a set of practical tests for dosimetry calculations in radiotherapy, defined in a dedicated technical document, TECDOC 1583 [9].

This document has been used as the basis to conduct the dosimetric audit of TPSs in Serbia. The methodology of this audit focuses on dosimetry part of the treatment planning and delivery processes. An anthropomorphic phantom is used with a set of clinical test cases prepared by the IAEA, covering a range of typical clinical radiation techniques in 3D CRT. The audit methodology verifies the chain in external beam radiotherapy workflow, from patient data acquisition to treatment planning and dose delivery.

## Methods

The audit was conducted in three out of six radiotherapy departments in Serbia, i.e. the Institute of Oncology of Vojvodina in Sremska Kamenica, the Clinical Centre in Niš and the Institute of Oncology and Radiology of Serbia in Belgrade. Hospitals where audit was not conducted either did not have computerized TPS or their treatment machines were out of working order during the auditing period. The audit programme required two days per hospital to be carried out.

### Phantom

**Figure 1:**
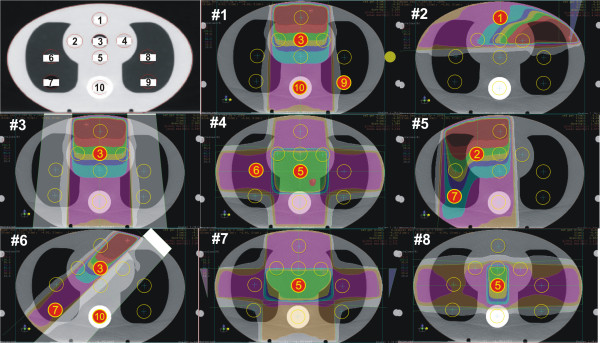
Position of measurement points in CIRS thorax phantom and beam geometry, measurement points and sample dose distribution for eight test cases.

The choice of the phantom for this study was based on the following considerations: minimal restricted flexibility, easy handling and capability to perform all dosimetric and anatomical test cases. From the comparison of different phantoms for clinical commissioning of TPSs following an IAEA protocol given in TECDOC 1583 [9], it was chosen that the clinical test case measurements will be conducted on the semi-anthropomorphic phantom CIRS Thorax 002 LFC (CIRS Inc., Norfolk, Virginia). The phantom is elliptical in shape (30 cm long x 30 cm wide x 20 cm thick) and represents an average human torso in proportion, density and two-dimensional structure. The body of the phantom is made of plastic water, lung and bone sections containing 10 holes to hold interchangeable rod inserts for an ionization chamber. The holes are numbered as shown in Figure 1. The phantom was supplemented with a set of four reference plugs with well-defined relative electron densities (muscle, bone, lung and adipose equivalent tissue).

The phantom was scanned in each hospital using a CT. Dose measurements were performed by placing the calibrated ionization chamber into the different holes in the phantom. The scanning procedure and that for the dose measurements are described in the sections below.

### CT calibration

The purpose of this test was to verify the Hounsfield units (HU) to relative electron density (RED) conversion curve stored in the TPSs. The phantom was scanned twice in each hospital using a CT. For the first scan the reference plugs with different known material properties were inserted into the holes in the phantom to check CT numbers to the RED conversion curve. Recommended arrangement of the reference plugs for the first CT scan were: hole 2- muscle reference plug, hole 4 - adipose reference plug, hole 5 - syringe filled with water, hole 6 - lung reference plug, hole 7 - should be empty to represent air and hole 10 - bone reference plug.

The second scan was done without reference plugs and was used for the planning of clinical test cases as defined in the TPS audit exercise. During second scan all holes were filled with appropriate rod inserts. The local scanning protocol was used, and the scanning parameters for both scans were kept the same. The acceptance criteria for the difference between the stored and measured values of CT numbers for the same RED was ± 20*HU*[8].

### Clinical test cases

A set of clinical test cases was created to verify a range of basic treatment techniques applied in the clinical practice. The beam geometry and sample dose distribution are shown in Figure 1. The detailed description of test cases is given in the IAEA TECDOC 1583 [9]. The measurement points for each case were selected to avoid high dose gradients and measurements in the penumbra region. The total number of measurement points in eight test cases was fifteen. The same set of clinical test cases was applied in all three hospitals. The dose calculations were performed for each available algorithm based on the grid size normally used in the hospital’s clinical practice.

### Treatment planning systems

Two different calculation algorithms implemented on three CMS XiO (Elekta CMS Software, St. Louis, Missouri) versions 4.33, 4.40 and 4.60 TPSs were investigated. All three hospitals use inhomogeneity corrections in clinical practice. The full description of implemented calculation algorithms are beyond the scope of this paper and can be found elsewhere [10-13]. The algorithms in this study have been divided into two groups:

Type (a) algorithms. Model based algorithms where changes in lateral electron and photon transport are not modeled (no lateral transport). Such algorithms use a pencil beam convolution model and primarily equivalent path length corrections to account for inhomogeneities.

Type (b) algorithms. Model based algorithms where changes in lateral electron and photon transport are approximately modeled (with lateral transport). These algorithms use a point kernel convolution/superposition model and account for the density variation in 3D.

### Measurements

CT scanners used in the study were Somatom 4 Plus (Siemens, Erlangen) and Light speed RT (General Electric Inc., Fairfield, Connecticut). Dose measurements were performed in three hospitals using different linear accelerators with nominal photon energies of 6 and 15 MV from the Varian Clinac 2100 series (Varian Medical Systems, Palo Alto, California); 6 and 15 MV beams from Siemens Oncor accelerators (Siemens Medical Solutions, Erlangen) and 6 and 15 MV beams from Elekta Synergy accelerators (Elekta Oncology Systems, Crawley). The photon beams were divided according to the energy into two groups: lower energy X-ray (6 MV) and higher energy X-ray (15MV) beams. In two institutions Farmer type chambers FC65-G (IBA Dosimetry, Schwarzenbruck, Germany) were used while in one institution ionisation chamber NE 2571 (Nuclear Enterprise Technology, U.K) was employed. In all measurements Dose 1 (IBA Dosimetry, Schwarzenbruck, Germany) electrometer was used. All chambers and the electrometer were calibrated by the national secondary standards dosimetry laboratory. For all measurement points, the absorbed dose to water was determined from ionization chamber measurements using the IAEA TRS 398 dosimetry code of practice [14]. When measuring in lung and bone equivalent materials it is assumed that the doses are measured in small water cavities within these materials. Since these small cavities have not been outlined on CT slices during dose calculations, the reported doses in these materials have a larger uncertainty than those in plastic water. The impact of water cavities has been estimated to increase the calculated dose by up to 2% for lung equivalent material and up to 0.3% for bone equivalent material in worst case scenarios.

### Analysis of the results

For the evaluation of the measured (D_meas_) and TPS calculated (D_cal_) values the criteria specified in IAEA TRS 430 were employed. However, due to the limited number of available positions for dose measurements in the phantom and for better consistency in the interpretation of the results for the various points the dose differences were normalized to the dose measured at the reference point for each test case, i.e. the following equation was used:

$$\delta \left(\%\right)=100x\frac{\left(D_{\text{calc}}-D_{\text{meas}}\right)}{D_{\text{meas},\text{ref}}}$$

where D_meas, ref_ is the dose value measured at the reference point. This reference point is a point that is expected to have received 2 Gy, and it was specified for each test case. For multiple beam combination the difference between measured and calculated dose values for selected beam should be related to the dose measured at the reference point for the corresponded beam. The agreement criteria for each test case were determined according to the complexity of the test case geometry.

## Results

### CT to RED conversion

**Figure 2:**
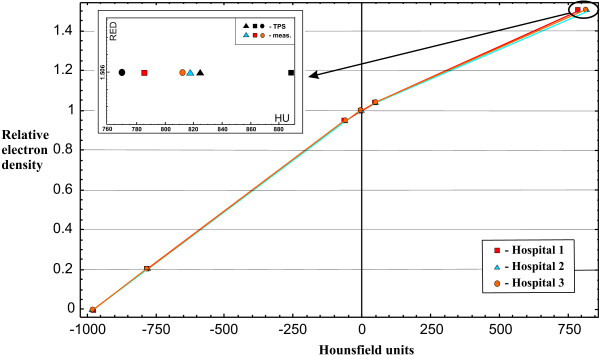
CT calibration curves measured with CIRS phantom at three different hospitals.

All systems reviewed in this audit had generic or TPS manufacturer supplied CT to RED conversion curves. Based on the measurements, we concluded that there were differences of 6-12% in the region of higher electron densities in two out of three cases (Figure 2). However, it was estimated that this difference in relative electron density affects dose calculation accuracy less than 2% [9,15]. In Figure 2 we also presented a zoom at high density region with TPS data added.

### Clinical test cases

**Figure 3:**
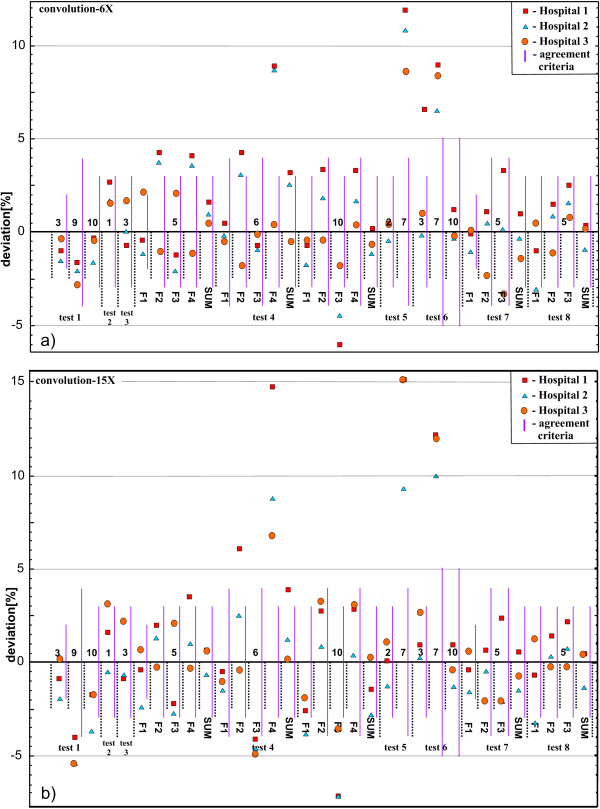
**Difference between measured and calculated point doses for each test case for model based algorithms - no lateral transport.** (**a**) 6 MV photon beams, (**b**) 15 MV photon beams.

**Figure 4:**
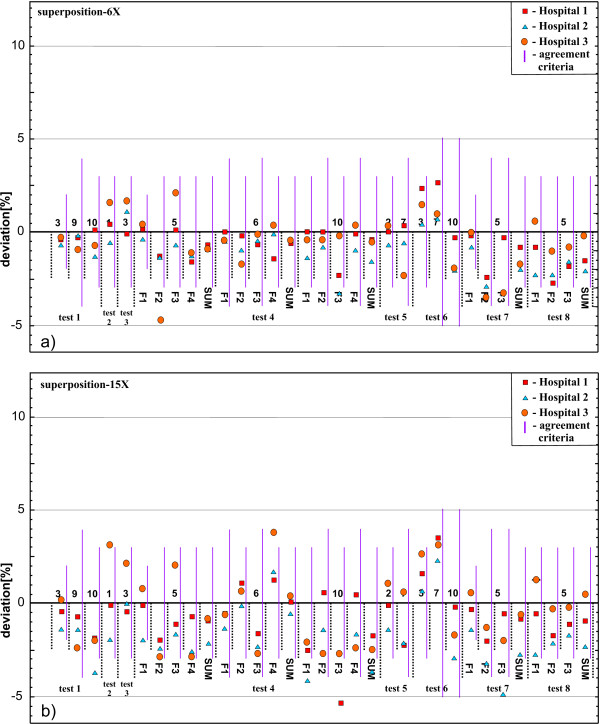
**Difference between measured and calculated point doses for each test case for model based algorithms with lateral transport.** (**a**) 6 MV photon beams, (**b**) 15 MV photon beams.

The differences between the measured and calculated doses for the various measurement points and test cases for all three hospitals are presented in Figures 3 and 4. The results are grouped according to the energies and the calculation algorithms implemented (with or without lateral transport). Also the value of the agreement criteria for each measurement point and their sum, for the points where there are contributions from a several beams coming from various directions, is shown as a thick purple line.

The differences in the results between centres with similar calculation algorithms could be partly attributed to model beam fitting process and CMS XiO TPS limitations to model different accelerator’s heads.

The largest deviations were observed in the customised blocking test **(test 5)** in point 7, which was located within the lung equivalent material, see Figure 3. The differences up to 9% for lower energy beams and up to 15% for higher energy beams were found for the type (a) algorithms. For the type (b) algorithms (Figure 4) the results for both groups of beam energies were within the agreement criteria.

The irregular L-shaped field test **(test 6)** had three measurement points: one in plastic water (point 3), one in the lung equivalent material (point 7) and one in the bone equivalent material (point 10). Large deviations up to 9% for lower energy beams and up to 12% for higher energy beams were observed in point 7 for the algorithms type (a), as can be seen in Figure 3. Type (b) algorithms showed results within the agreement criteria (Figure 4).

The four-field box test **(test 4)** had three measurement points: one at the isocentre in plastic water (point 5), one in the lung equivalent material on the central axis of lateral beams (point 6) and one in the bone equivalent material on the central axis of vertical beams (point 10). The deviations outside agreement criteria were found for points 6 (lung) and 10 (bone) for type (a) algorithms for all energies (Figure 3). The differences up to 9% for lower energy beams and up to 14.8% for higher energy beams were found in point 6 (lung). The dose in point 10 (bone) was underestimated by 6% for lower energy beams and 7.2% for higher energy beams. Type (b) algorithms yielded results within the agreement criteria (Figure 4).

Before the audit, all three centers used algorithms type (a) for dose calculation in treatment plans for all patients. During the comparison of both algorithm types in clinical cases the largest deviations were observed for lung tumor patients treated with higher energy beams. It was noticed that high energy lung treatment plans calculated by algorithms type (a) may result in reduction of PTV volume covered by 95% isodose by up to 18%. Due to this problem, the change in clinical practice was introduced after the audit in all three centers and all dose calculations in the thorax region are performed with type (b) algorithm.

Also, the verification of basic dosimetry data input into TPS was done. After the review of the dosimetric data entered into the local TPS, it was found that the head scatter factors for a 15 MV photon beam in one of the hospitals were not correct and new measurements with a mini phantom were performed to correct this problem. In another hospital, a mistake in a wedge factor entry for a high energy beam and 15 degree hard wedge was discovered for the field 15x15 cm^2^ and it was corrected.

The TPS data review within this audit appeared to be an excellent opportunity to spot any errors in the local TPS data and correct them.

## Discussion

An adjustment in CT numbers to the RED conversion curve was needed in two out of three TPSs in the high density region according to criteria from TRS 430. The differences of 6-12% were found in the region with densities above that for water. However, the magnitude of the error in the calculated dose due to this difference was estimated to be less than 2% for the 6 MV photon beam passing through 5 cm thick material with the RED of 1.5 [9,15]. Therefore, the adjustments were not carried out.

Although the TPS algorithm testing was not the purpose of the audit, several general conclusions could be drawn. The systematic dose overestimation by up to 15% for the type (a) calculation algorithms was recorded for all measurement points located inside the lung equivalent material. It was observed that the range of deviations was related to the beam energy, i.e. larger deviations were observed for the higher beam energy [16]. Doses inside the bone equivalent material were underestimated by up to 7% for both 6 MV and 15 MV beams for both algorithm types [17]. In general, the type (a) algorithms are not adequate for dose calculations in the presence of and inside low density inhomogeneities, while the type (b) algorithms showed good results for all test cases.

Accordingly, a comparison of calculation for different clinical lung treatment plans was carried out. Patient plans were calculated by both algorithms, but irradiated according to the results of the advanced type (b) algorithm. The overall treatment time calculated with algorithm type (b), was 5–7% longer in comparison to the calculation of algorithm type (a), and the coverage of PTV, in terms of 95% isodose, was better (up to 18%). That means that the plan calculated by the simpler algorithm was actually overestimating the dose to be delivered, in reality leading to the underdosage of the target volume. This applies both to lower energy and even more to higher energy beams.

Differences between doses calculated with algorithms type (a) and (b) are primarily due to changes in electron transport in the lungs, which is not adequately taken into account by algorithms type (a). Many papers have demonstrated that simplistic algorithms can overestimate the dose at tumor lung boundary which may be misleading clinically and even contra-indicated [18-21].

Following findings from the audit, and recommendations from the literature [11], the preferred beam energy for planning the lung tumors, was moved from higher to lower energies in most thorax cases in the audited clinics. In some cases were used combinations of higher and lower energy beams due to better dose distribution.

Transition to more advanced algorithms provides a better consistency between the reported and actually delivered doses, which opens opportunities for establishing a more precise dose-volume relationship for tumours and normal tissues [22].

The range of the dose deviations that occurred in this audit exercise reflects the relative dosimetric accuracy of the treatment planning process from CT scanning to the dose delivery [23]. The accuracy of the dose calculation algorithm is one of the main factors affecting the overall uncertainty of the dose delivered to the patient, and it is very important to perform various tests to better understand the TPS limitations. The test cases presented within this audit proved to be useful to verify the TPS calculations with measurements and to estimate the magnitude of algorithm limitations in situations close to clinical settings, however, it should be understood that the type and number of tests performed should depend on the local practice of a particular institution.

The users of different TPSs may utilize the final results of tests as a reference data for the ongoing periodic QA checks. However, it is important to emphasize that the final dose calculation results may be affected by different factors, such as the dose calculation grid, inadequacies in input data, the choice of phantom used, and others.

The ionization chamber dosimetry with a limited number of points has some restrictions as the results may depend on the selection of individual points. However, the end-to-end approach is considered adequate for the evaluation of the overall quality of the dose calculations and to explore the limitations of the TPS [9,24]. Some aspects such as the penumbra widening in low density materials at higher energy beams (test 1, point 9) may be better explored using film dosimetry but this was beyond the objectives of this study [23].

Another advantage of the TPS audit is that it can be performed in a reasonable amount of time in hospitals with high patient load and limited staff and enables the user to have his/her work evaluated by an independent peer reviewer.

## Conclusions

The methodology described in the IAEA TECDOC 1583 was used to perform TPS audits in three radiotherapy centers of Serbia. A few discrepancies in basic dosimetric data were discovered and corrected. The results also indicated the shortcomings of type (a) treatment planning algorithms and, therefore the transition to more advanced algorithms, type (b), was implemented [25]. In addition, it was decided to consistently plan future lung cancer treatments with lower energy photon beams for which the TPS dose calculations are more accurate compared to those for higher energy beams. The audit could also help the users to appreciate the properties, qualities and operational characteristics of treatment planning systems and to better understand their limitations.

## Competing interests

The authors declare that they have no competing interests.

## Authors’ contributions

LR: substantial contributions to conception and design, acquisition of data, treatment planning, analysis and interpretation of data, writing the draft of the manuscript, revising it critically for important intellectual content. BP: analysis and interpretation of data, substantial contributions to conception and design, revising it critically for important intellectual content, have given final approval of the version to be published. MB: treatment planning of test cases, revision of manuscript. MT: treatment planning of test cases, revision of manuscript. OČ: acquisition of data, revision of manuscript. EG: substantial contributions to conception and design, acquisition of data, analysis and interpretation of data, drafting the manuscript, revising it critically for important intellectual content, have given final approval of the version to be published. JI: substantial contributions to conception and design, drafting the manuscript, revising it critically for important intellectual content, have given final approval of the version to be published. All authors read and approved the final manuscript.
